# Crucial roles of calcium ATPases and phosphoinositides: Insights into pathophysiology and therapeutic strategies

**DOI:** 10.1016/j.mocell.2025.100254

**Published:** 2025-07-11

**Authors:** Hyun-Oh Gu, Seung Wan Noh, Ok-Hee Kim, Byung-Chul Oh

**Affiliations:** 1 Department of Health Science and Technology, Gachon Advanced Institute for Health Science and Technology, Gachon University, Incheon 21999, South Korea; 2 Department of Physiology, Lee Gil Ya Cancer and Diabetes Institute, College of Medicine, Gachon University, Incheon 21999, Korea

**Keywords:** Ca^2+^-ATPases, Ca²⁺-phosphoinositide interaction, Calcium signaling, Intracellular Ca²⁺ homeostasis, Pathophysiology

## Abstract

Calcium (Ca²⁺) serves as a pivotal intracellular messenger, influencing a diverse array of cellular processes, including muscle contraction, neurotransmission, and hormone secretion. It also plays a critical role in the regulation of gene expression. Intracellular Ca²⁺ levels are stringently controlled and maintained within a narrow physiological range, primarily by plasma membrane Ca^2+^-ATPases, sarco-/endoplasmic reticulum Ca^2+^-ATPases, and secretory pathway Ca^2+^-ATPases. These ATPases orchestrate the influx, efflux, and sequestration of Ca²⁺ across cellular compartments, thereby ensuring cellular functionality and survival. This review delves into the intricate interplay between Ca²⁺ and phosphoinositides, essential lipid signaling molecules that modulate Ca^2+^-ATPase activities and link Ca²⁺ signaling to a wide range of cellular functions. By examining the molecular dynamics of Ca^2+^-ATPases and their regulatory interactions with phosphoinositides, we discuss their roles under both physiological and pathological conditions, highlighting how disturbances in these interactions contribute to disease. Furthermore, we explore the potential of targeting these Ca²⁺ regulatory mechanisms as a therapeutic strategy for diseases characterized by Ca²⁺ dysregulation, providing insights into future research directions and clinical applications.

## INTRODUCTION

Ca²⁺ plays a crucial role as an intracellular messenger, influencing diverse biological processes such as muscle contraction, neurotransmission, and hormone secretion, while also regulating enzyme activity and gene expression (Cahalan and Chandy, 2009, Clapham, 2007). Intracellular Ca²⁺ levels are meticulously regulated within a narrow range of 50 to 100 nM, which contrasts sharply with the much higher extracellular concentrations of 1 to 2 mM. This steep gradient is crucial for activating a variety of cellular functions and is maintained by an intricate network of transporters and channels, including plasma membrane Ca^2+^-ATPases (PMCAs), sarco-/endoplasmic reticulum Ca^2+^-ATPases (SERCAs), and secretory pathway Ca^2+^-ATPases (SPCAs) (Arruda and Hotamisligil, 2015, Bagur and Hajnóczky, 2017).

These transport systems regulate Ca²⁺ influx and efflux and also facilitate its sequestration across cellular compartments, ensuring the dynamic regulation of Ca²⁺ required for cellular function and viability. PMCAs expel Ca²⁺ from the cytosol into the extracellular space, SERCAs reuptake Ca²⁺ into the ER, which is crucial for restoring cytosolic Ca²⁺ levels following cellular activation (Chemaly et al., 2018), and SPCAs transport Ca²⁺ into the Golgi apparatus for critical post-translational modifications (Rahate et al., 2020, Wuytack et al., 2002).

An additional layer of Ca²⁺ regulation involves phosphoinositides (PIPs), which play significant roles in cellular signaling pathways. PIPs, especially phosphatidylinositol 4,5-bisphosphate (PI(4,5)P_2_) (Bruce, 2018), are integral to the activation of plasma membrane Ca²⁺ channels and regulation of intracellular Ca²⁺ levels. They interact with various Ca²⁺ transporters to modulate their activity, linking Ca²⁺ signaling to other cellular processes. For example, PI(4,5)P_2_ enhances PMCA activity by increasing its affinity for Ca²⁺ and ATP, thus promoting efficient Ca²⁺ extrusion and maintaining low intracellular Ca²⁺ concentrations (Moccia et al., 2023).

Furthermore, intracellular Ca²⁺ can form complexes with PIPs, thereby affecting the functions of key signaling molecules. Elevated intracellular Ca²⁺ levels lead to the formation of Ca²⁺-PIP complexes, which can inhibit the interaction of proteins with their respective PIP-binding domains, thereby affecting signal transduction pathways and membrane localization of proteins involved in critical cellular functions (Kang et al., 2017). This interplay between Ca²⁺ and PIPs is crucial for maintaining cellular signaling integrity and has been implicated in various pathological conditions, highlighting potential therapeutic targets within these regulatory mechanisms.

Understanding the molecular dynamics of Ca^2+^-ATPases, their interactions with PIPs, and the implications of these interactions under physiological and pathological conditions provides a comprehensive framework for exploring therapeutic strategies to modulate Ca²⁺ homeostasis. This review delves into the mechanisms of Ca²⁺ regulation through Ca^2+^-ATPases and PIPs, emphasizing their roles in health and disease, and discusses potential interventions targeting these pathways.

### Intracellular Ca^2+^ Dynamics and Master Regulators of Ca²⁺ Transport Systems

Ca²⁺ is the most prevalent divalent cation in the human body and serves as a critical intracellular messenger involved in numerous cellular functions, including muscle contraction, neurotransmission, and hormone secretion (Cahalan and Chandy, 2009, Clapham, 2007). To maintain intracellular Ca²⁺ homeostasis, a tightly regulated system of transporters and channels orchestrates Ca²⁺ influx, efflux, and sequestration across cellular compartments. The concentration gradient between the intracellular (50-100 nM) and extracellular (1-2 mM) environments is essential for activating a variety of cellular processes (Arruda and Hotamisligil, 2015). This regulation is achieved through a sophisticated network of Ca²⁺ transporters, including PMCAs, SERCAs, and SPCAs, each playing distinct roles in Ca²⁺ sequestration and mobilization (Bagur and Hajnóczky, 2017). PMCAs actively expel cytosolic Ca²⁺ into the extracellular space, SERCAs facilitate Ca²⁺ reuptake into the ER to restore cytosolic Ca²⁺ levels after cellular activation (Chemaly et al., 2018), and SPCAs mediate Ca²⁺ and manganese ion (Mn²⁺) transport into the Golgi apparatus, which is essential for post-translational modifications, such as glycosylation (Rahate et al., 2020, Wuytack et al., 2002). Additionally, depletion of ER Ca²⁺ is detected by stromal interaction molecule 1 (STIM1) and STIM2, which activate ORAI Ca^2+^ release-activated Ca^2+^ modulator (ORAI) and transient receptor potential C-type channels to facilitate store-operated Ca²⁺ entry (SOCE), a critical replenishment mechanism (Brandman et al., 2007). Further regulation is provided by various Ca²⁺-permeable ion channels, including TRP channels, purinergic ionotropic receptors, voltage-activated Ca²⁺ channels, and the mitochondrial Na⁺/Ca²⁺/Li⁺ exchanger (NCLX), which together maintain the dynamic regulation of Ca²⁺ across cellular compartments (Trebak and Kinet, 2019).

**Fig. 1:**
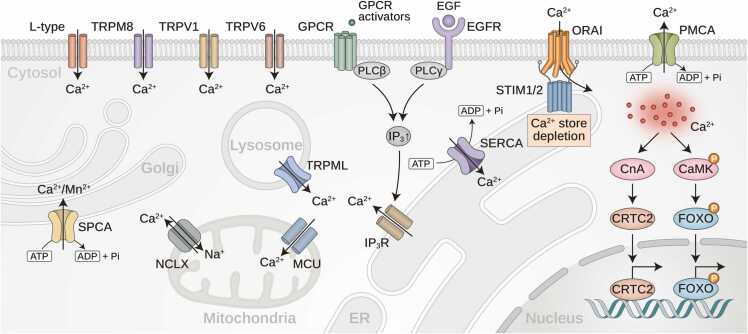
Regulation of intracellular Ca^2+^ signaling and homeostasis across various cellular compartments. Ca^2+^ homeostasis within the cytoplasm, ER, and mitochondria is regulated by transporters and pumps that include PMCAs, SERCAs, and mitochondrial NCLX. Additional Ca^2+^ influx is mediated by plasma membrane channels, including TRP channels and purinergic ionotropic receptors, which are activated during GPCR signaling. GPCRs respond to agents like glucagon and catecholamines, or T-cell receptors respond to specific antigens, activating PLC to produce IP_3_ and DAG. IP_3_ stimulates the release of Ca²⁺ from ER stores by binding to IP_3_Rs. This depletion of ER Ca²⁺ is detected by STIM1 and STIM2, activating ORAI1 proteins on the plasma membrane and inducing SOCE. IP_3_Rs also mediate the transfer of Ca²⁺ to mitochondria via the MCU at mitochondrion-associated membranes, enhancing ATP production. Concurrently, GPCR stimulation can increase cAMP levels, activating protein kinase A, which phosphorylates and activates IP_3_Rs. Elevated cytosolic Ca²⁺ levels activate CaMK and CnA, influencing downstream targets, including transcription factors such as FOXO, CREB, and NFAT and CREB-regulated transcription coactivator 2.

Upon hormonal stimulation, G protein-coupled receptors (GPCRs) activate downstream signaling cascades that influence intracellular Ca²⁺ dynamics. For example, catecholamines stimulate adenylate cyclase via Gs alpha subunit proteins, increasing cAMP levels and activating protein kinase A, which in turn phosphorylates inositol trisphosphate receptors (IP_3_Rs) to promote Ca²⁺ release from the ER (Foskett et al., 2007). Additionally, phospholipase C (PLC) hydrolyzes PIP_2_ to generate DAG and IP_3_, which activates IP_3_Rs on the ER to release Ca^2+^ (Foskett et al., 2007, Streb et al., 1985). IP_3_ is further phosphorylated into diverse IPs and PP-IPs, forming a signaling network that regulates key cellular functions (Kim et al., 2024). Spatial Ca²⁺ signaling is governed by stimulus intensity—weak signals induce localized Ca²⁺ puffs, while strong stimuli trigger global Ca²⁺ waves. PIP_2_ primes IP_3_Rs but is depleted by GPCR activation, resetting IP_3_R sensitivity and regulating the shift from local to global Ca²⁺ signaling (Ivanova et al., 2024). Elevated intracellular Ca²⁺ levels activate Ca²⁺-sensitive signaling molecules, such as calmodulin (CaM), calcineurin, and protein kinase C (Choi et al., 1994, Clapham, 2007), which regulate key transcription factors, such as forkhead box O (FOXO), cAMP response element binding (CREB), p38, nuclear factor-kappa B, and c-Jun N-terminal kinase (JNK), influencing metabolism, inflammation (Rhee et al., 2024), and cellular survival (Zhang et al., 1995). Additionally, calcineurin dephosphorylates transcriptional regulators, such as CREB-regulated transcription coactivator 2, myocyte enhancer factor 2, nuclear factor of activated T cells c, and transcription factor EB, allowing its translocation into the nucleus to initiate gene expression programs crucial for metabolic regulation, immune responses, lysosome biogenesis, and autophagy (Fig. 1) (Choi et al., 1994).

Mitochondrial Ca²⁺ transport systems, including the mitochondrial Ca²⁺ uniporter (MCU) and NCLX, regulate mitochondrial Ca²⁺ levels essential for bioenergetics and metabolic regulation (De Stefani et al., 2016). Unlike Ca²⁺-ATPases, which actively transport Ca²⁺ via ATP hydrolysis, mitochondrial Ca²⁺ transporters rely on preexisting ion gradients to facilitate Ca²⁺ flux, ensuring efficient energy production (Li et al., 2023, Wang et al., 2025). The distinct structural characteristics of PMCAs, SERCAs, and SPCAs revealed by advanced techniques, such as cryoelectron microscopy (cryo-EM) and X-ray crystallography, underscore their evolutionary adaptations for specialized Ca²⁺ handling in different organelles (Bagur and Hajnóczky, 2017, Stokes and Green, 2003). Given their essential roles in Ca²⁺ homeostasis, dysfunction in these transport systems has been implicated in various pathological conditions, making them potential therapeutic targets for disorders associated with Ca²⁺ dysregulation.

### Molecular Regulation of SERCA: Linking ATP Dependency to ER Stress and Metabolic Disease

**Fig. 2:**
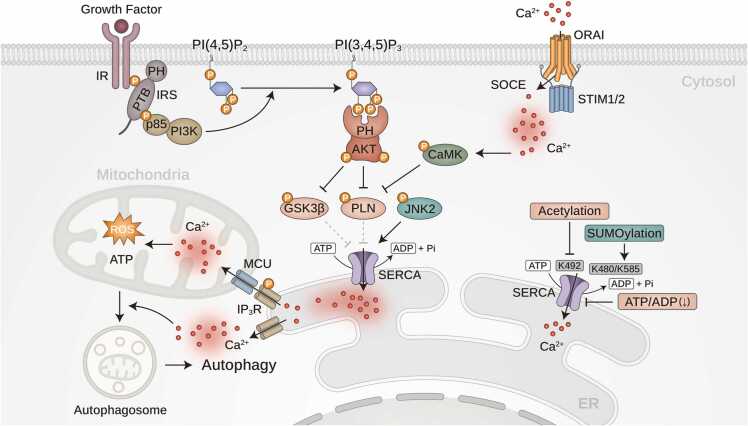
Regulation of SERCA activity by post-translational modifications. Growth factor signaling activates PI3K/AKT pathway, leading to the production of PI(3,4,5)P_3_ and subsequent AKT activation. Activated AKT phosphorylates PLN, relieving its inhibitory effect on SERCA and enhancing Ca²⁺ reuptake into the ER. Additionally, AKT modulates SERCA activity through the phosphorylation of GSK3β, further regulating Ca^2+^ homeostasis. SOCE, mediated by STIM1/2 and ORAI channels, facilitates extracellular Ca^2+^ influx into the cytosol. The resulting increase in Ca²⁺ activates CaMK, which phosphorylates PLN to further enhance SERCA function and prevent intracellular Ca^2+^ overload. In addition, c-Jun N-terminal kinase 2 (JNK2) directly phosphorylates SERCA, increasing its activity. Post-translational modifications, including SUMOylation and acetylation, further fine-tune SERCA function, while ATP depletion (↓ ATP/ADP) disrupts Ca^2+^ homeostasis and contributes to autophagy. Mitochondrial Ca^2+^ uptake via the MCU is also depicted, along with the generation of ROS and production of ATP. The intricate interplay between ER Ca^2+^ regulation, mitochondrial function, and autophagy highlights the critical role of Ca^2+^ homeostasis in metabolism and cellular stress responses.

SERCA is an ATP-dependent pump essential for maintaining intracellular Ca^2+^ homeostasis by actively transporting Ca^2+^ from the cytosol to the sarcoplasmic reticulum (SR) or ER against steep Ca^2+^ gradients, with ER Ca^2+^ concentrations 1000 to 10,000 times higher than cytosolic levels (Tadini-Buoninsegni et al., 2018). This energy-intensive process renders SERCA highly sensitive to ATP depletion, which impairs Ca^2+^ sequestration and leads to ER Ca^2+^ depletion, cytosolic Ca^2+^ overload, and disrupted Ca^2+^ signaling (Gonnot et al., 2023). These disruptions induce ER stress and activate the unfolded protein response, resulting in apoptosis (Viskupicova and Rezbarikova, 2022). Simultaneously, excess cytosolic Ca^2+^ is taken up by mitochondria, causing mitochondrial dysfunction, production of reactive oxygen species (ROS), and activation of apoptotic pathways (Curman et al., 2024, Gonnot et al., 2023) (Fig. 2).

SERCA activity is tightly regulated by multiple mechanisms, including post-translational modifications (PTMs) and interactions with regulatory proteins, to ensure precise Ca^2+^ signaling under various physiological conditions. PTMs significantly influence SERCA function, with kinase-mediated phosphorylation categorized into activity-enhancing and activity-suppressing modifications. AKT regulates SERCA function by phosphorylating phospholamban (PLN), which alleviates the inhibitory effect of PLN on SERCA, thereby enhancing Ca²⁺ reuptake into the ER (Bhupathy et al., 2007). This process ensures efficient Ca^2+^ homeostasis, particularly during conditions requiring rapid Ca²⁺ clearance from the cytosol, such as cardiac muscle relaxation or cellular signal adaptation. When AKT is activated, PLN is phosphorylated, leading to increased SERCA activity and improved Ca²⁺ sequestration. Conversely, when AKT is inhibited or inactivated, PLN remains unphosphorylated, sustaining its inhibitory effect on SERCA and reducing Ca²⁺ uptake into the ER, which may contribute to prolonged cytosolic Ca²⁺ elevation and altered cellular responses. Similarly, activity-suppressing phosphorylation of SERCA2A at serine 663 by glycogen synthase kinase 3 beta (GSK3β) reduces SERCA2 activity, leading to decreased Ca^2+^ uptake, cytosolic and mitochondrial Ca^2+^ overload, and increased cell death in ischemic heart disease and reperfusion injury (Gonnot et al., 2023). Inhibiting GSK3β or preventing serine 663 phosphorylation restores SERCA2 efficiency, alleviates Ca^2+^ overload, and protects against myocardial infarction, thus offering a promising therapeutic strategy (Gonnot et al., 2023). Conversely, activity-enhancing phosphorylation by JNK2 boosts SERCA2 activity and enhances ER Ca^2+^ pump function (Yan et al., 2021), although it may increase arrhythmic risk by altering the balance between SR Ca^2+^ leakage and storage, particularly in aged and alcohol-exposed hearts. Phosphorylation of SERCA2a at threonine 484 via the AKT-SK1/SK2 pathway enhances Ca^2+^ reuptake. Impairment of this phosphorylation has been implicated in early diabetic cardiomyopathy (Quan et al., 2022). Other PTMs, such as SUMOylation at lysine residues 480 and 585, preserve SERCA2a activity and stability, with reduced SUMO1 levels contributing to heart failure (Gotthardt and Lehnart, 2024, Kho, 2023). Acetylation at K492, which is mediated by p300 and linked to reduced SIRT1 levels, impairs SERCA2a by disrupting ATP binding. Pharmacological activation of SIRT1 can reverse this effect, improving cardiac performance (Gorski et al., 2019).

Most importantly, small membrane-bound peptides, such as PLN, sarcolipin, myoregulin, dwarf open reading frame (DWORF), and sarcolamban A and B (SCLA/SCLB), further regulate SERCA activity and Ca^2+^ homeostasis in muscle and cardiac tissues. PLN, sarcolipin, and myoregulin inhibit SERCA, whereas DWORF enhances SERCA activity by displacing the inhibitory peptides (Fisher et al., 2021). In Drosophila, SCLA and SCLB inhibit SERCA, and neprilysin 4 hydrolyzes these peptides to regulate SERCA activity and ensure proper Ca^2+^ homeostasis. Similar regulatory mechanisms have been observed in humans (Schiemann et al., 2022). SERCA dysfunction due to ATP depletion is implicated in numerous diseases, including diabetes, where it exacerbates insulin resistance and β-cell dysfunction (Kho et al., 2012, Viskupicova and Rezbarikova, 2022), heart failure, where impaired SERCA2a disrupts Ca^2+^ reuptake and cardiac contractility (Kho et al., 2012), and neurodegenerative diseases, such as Alzheimer’s and Parkinson’s, where it contributes to excitotoxicity, oxidative stress, and neuronal death (Rakovskaya et al., 2023). By serving as a central regulator of Ca^2+^ homeostasis, ATP dependency and post-translational regulation of SERCA establish critical links between metabolic stress, Ca^2+^ signaling, and disease pathogenesis (Gonnot et al., 2023).

### Molecular Regulation of PMCA and Pathophysiological Implications

**Fig. 3:**
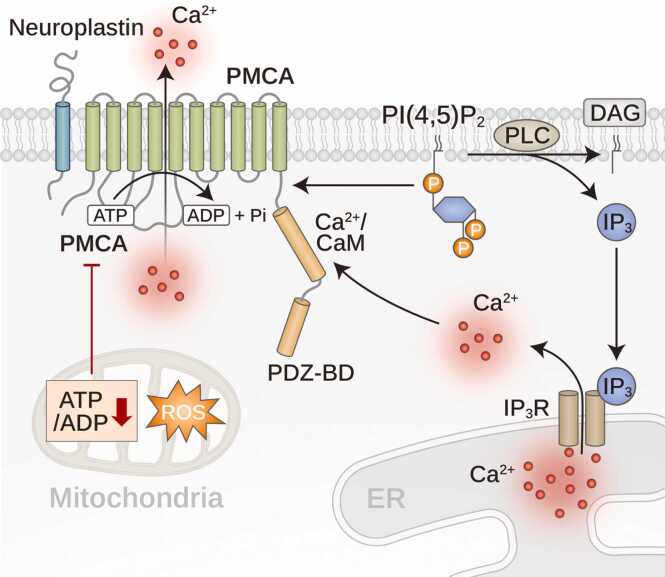
Regulation of PMCA activity. PMCA, a P-type ATPase, is essential for extruding cytosolic Ca²⁺ to maintain intracellular Ca²⁺ homeostasis. PMCA has a pivotal role in modulating Ca²⁺ signaling and preventing cytotoxic Ca²⁺ overload. This ATP-driven pump is regulated through various mechanisms. It is activated by CaM binding, phosphorylation events, and interactions with PIPs and PDZ-domain-containing proteins. Neuropilin aids in stabilizing PMCA on the membrane, thereby enhancing its function. PMCA activity can be inhibited by reduced ATP/ADP ratios due to mitochondrial dysfunction or elevated levels of ROS, impairing its capacity to manage Ca²⁺ levels effectively. Additionally, PI(4,5)P_2_ interaction increases the affinity of PMCA for Ca²⁺ and ATP, and protects it from PIP_2_ degradation by PLC. This degradation typically leads to the production of IP_3_, which prompts Ca²⁺ release from the ER via IP_3_R. The intricate interactions between PMCA, PIPs, and ER Ca²⁺ release are vital for maintaining Ca²⁺ homeostasis, which is crucial for cellular signaling and survival.

To sustain vital functions, eukaryotic cells must maintain a low cytosolic Ca²⁺ concentration (∼50-200 nM), despite much higher extracellular levels (1-2 mM, up to 10 mM in seawater). To manage this steep gradient, cells utilize effective Ca²⁺ extrusion systems: the PMCA and NCX pumps in the plasma membrane (PM) and SERCA and SPCA pumps internally in the ER/SR and secretory pathways, respectively. These systems work together to finely tune Ca²⁺ levels, which are crucial for signal transduction and cellular processes, such as fertilization. Imbalances in these systems, which lead to Ca²⁺ overload, can trigger cell death. The PMCA pump, which is characterized by high Ca²⁺ affinity but low transport capacity, serves as a precise cytosolic regulator (Souto-Guevara et al., 2024) (Fig. 3). PMCA exists as 4 isoforms generated through alternative splicing, allowing for tissue-specific expression and diverse functions (Ribiczey et al., 2007). PMCA1 and PMCA4 play essential housekeeping roles and are ubiquitously expressed, whereas PMCA2 and PMCA3 are predominantly found in specialized tissues, where they fulfill their unique functional demands (Ribiczey et al., 2007). Neuroplastin and basigin are critical subunits that enhance PMCA efficiency, whereas PI(4,5)P_2_ is a major activator that stabilizes PMCA function and protects it from hydrolysis by PLC (Gong et al., 2018, Kowalski et al., 2023).

PIP_2_ contributes to approximately 50% of PMCA activity at rest, enhancing its affinity for Ca²⁺ and ATP (Kowalski et al., 2023). PMCAs protect PIP_2_ from degradation through active Ca²⁺ clearance and PIP2 binding, which reduces IP_3_ formation and intracellular Ca²⁺ release (Penniston et al., 2014). The conserved basic residue cluster in PMCA forms binding pockets for PIP_2_, contributing to the regulation of PI(4,5)P_2_-Ca²⁺ signaling at ER-PM junctions (Chang and Liou, 2016, Penniston et al., 2014). PI(4,5)P_2_, a major PMCA activator, increases the affinity of PMCA for ATP and enhances the binding of Ca^2+^/CaM to an autoinhibitory motif, thereby facilitating PMCA Ca^2+^ movement into the extracellular space (Bruce, 2018). Dysregulation of this system under conditions of ATP depletion or metabolic stress results in impaired PMCA activity, leading to intracellular Ca²⁺ overload, mitochondrial dysfunction, and cell death through necroptosis (Morgan and Kim, 2025) or apoptosis (Baggaley et al., 2008, Bruce, 2018, Castro et al., 2006) (Fig. 3).

PMCA dysfunction has been implicated in cancer, neurodegeneration, and metabolic disorders (Brini and Carafoli, 2009, Brini and Carafoli, 2011). In cancer cells, the Warburg effect supports PMCA activity by sustaining high ATP turnover, whereas glycolytic inhibitors disrupt PMCA function and induce selective apoptosis in glycolysis-dependent tumors (Boczek et al., 2021). Neurons are heavily reliant on PMCA to prevent excitotoxicity; as such, they experience synaptic dysfunction and progressive neuronal loss due to reduced PMCA activity in neurodegenerative conditions, including Alzheimer's and Parkinson's diseases (Gong et al., 2018). Regulatory mechanisms, including CaM activation and PIP stabilization, can enhance PMCA function, but are compromised by sustained ATP depletion (Hwang et al., 2021).

The isoform-specific roles of PMCA have also been linked to cancer progression, with altered expression patterns observed in gastric and colon cancers (Ribiczey et al., 2007). PMCA4b expression is significantly reduced in these cancers but increases upon the induction of differentiation, highlighting the potential of PMCA4b as a diagnostic and therapeutic target (Naffa et al., 2024). The diverse regulatory mechanisms of PMCA underscore its critical role in Ca^2+^ homeostasis, cellular signaling, and protection against Ca²⁺ overload, making it a promising focus for research and therapeutic intervention (Brini and Carafoli, 2011, Strehler et al., 2007).

### PIP Regulation of Ca^2+^-ATPase Activity

**Fig. 4:**
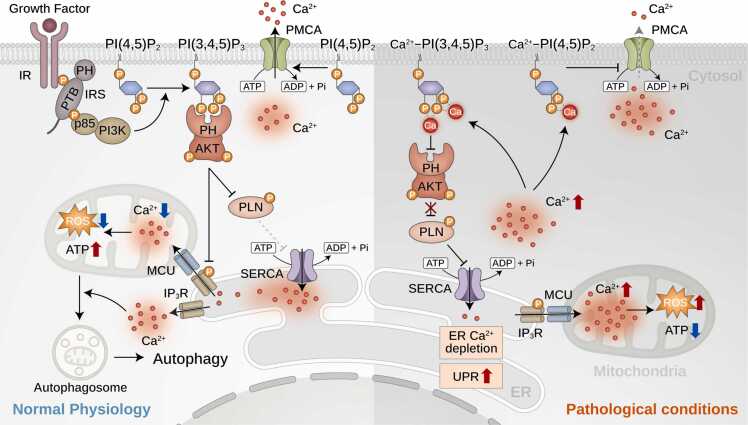
Phosphoinositide-dependent regulation of Ca^2+^-ATPases in physiological and pathological conditions. Growth factor signaling promotes the activation of PI3K, leading to the conversion of PI(4,5)P₂ into PI(3,4,5)P₃. PH domain-containing proteins, such as AKT, are recruited to the PM via interaction with PI(3,4,5)P₃, facilitating downstream signaling. AKT-mediated phosphorylation of PLN relieves its inhibitory effect on the SERCA, enabling efficient Ca^2+^ reuptake into the ER. Concurrently, PMCA expels cytosolic Ca²⁺, maintaining Ca^2+^ homeostasis. MCU facilitates Ca²⁺ uptake into the mitochondria, supporting ATP production while limiting the accumulation of ROS. Balanced Ca²⁺ dynamics sustain autophagy and metabolic function. Under pathological condition, elevated intracellular Ca²⁺ levels lead to the formation of Ca²⁺-PIP complexes (eg, Ca²⁺-PI(4,5)P₂ and Ca²⁺-PI(3,4,5)P₃), which impair PH domain interactions and prevent AKT recruitment to the PM. Consequently, PLN remains unphosphorylated, leading to SERCA inhibition, impaired ER Ca²⁺ reuptake, and ER Ca²⁺ depletion, which triggers the unfolded protein response. Additionally, Ca²⁺-PIP complex formation at the elevated intracellular Ca²⁺ also inhibits PMCA activity, exacerbating the accumulation of cytosolic Ca²⁺. Excessive mitochondrial Ca²⁺ levels induce mitochondrial dysfunction, increased oxidative stress, and reduced ATP generation, compounding cellular distress.

PIPs, notably PI(4,5)P_2_ and PI(3,4,5)P_3_, play important roles in modulating intracellular Ca^2+^ levels by interacting with Ca^2+^-ATPases, particularly PMCA and SERCA. PI(4,5)P_2_ is a potent PMCA activator (Krebs, 2022) (Fig. 4). It binds to PMCA at specific sites and dramatically increases the activity of the Ca^2+^ pump, essentially relieving the autoinhibitory conformation of the enzyme, similar to CaM. Binding of PI(4,5)P_2_ to the C-terminal CaM-binding domain increases its affinity for Ca^2+^ and ATP, enhancing its Ca^2+^ pumping activity (Carafoli, 1991). PIP₂ activates PMCA nearly as effectively as CaM. Activation of Gq-coupled receptors leads to PIP_2_ hydrolysis, temporarily inhibiting PMCA and resulting in increased intracellular Ca²⁺, which facilitates robust cellular signaling (Carafoli, 1991). Isoform-specific differences in PMCA regulation occur due to variations in CaM-binding and phospholipid-binding domains, with certain splice variants being more sensitive to PIP₂ modulation (Krebs, 2022).

PI(3,4,5)P_3_ primarily modulates Ca²⁺-ATPases via signaling pathways, notably phosphoinositide-3-kinase (PI3K)/AKT signaling. Activated AKT enhances SERCA activity in cardiac cells by phosphorylating PLN, relieving its inhibitory effect, and promoting Ca^2+^ uptake into the SR (Catalucci et al., 2009). Although direct activation of PMCA by PIP_3_ remains uncertain, its indirect influence through kinase pathways remarkably affects cellular Ca²⁺ management and survival. SERCA is indirectly regulated by PIPs because of its localization in the ER/SR membranes, which have low PIP content (Fig. 4). However, SERCA activity can be modulated by other acidic lipids, such as phosphatidylserine or phosphatidic acid, which stabilize its active conformation (Carafoli, 1991). Changes in local lipid composition during cellular stress or pathology may indirectly affect SERCA function through alterations in lipid-protein interactions (Aguayo-Ortiz and Espinoza-Fonseca, 2020). In cardiac cells, PIP3 signaling via PI3K/AKT indirectly enhances SERCA2a function through the phosphorylation of PLN, underscoring the importance of lipid-mediated signaling in SERCA regulation (Nagoshi et al., 2005, Nakadate et al., 2022). Beyond PIP-mediated regulation, intracellular Ca^2+^ homeostasis is critically governed by additional systems. The MCU facilitates Ca²⁺ uptake into the mitochondrial matrix during cytosolic Ca²⁺ spikes, thereby supporting ATP production and buffering cytosolic Ca²⁺; however, excessive uptake may trigger cell death via mitochondrial permeability transition (Rizzuto et al., 2012). CaM, a key Ca²⁺ sensor, activates transporters such as PMCA by relieving autoinhibition and regulates Ca²⁺/CaM-dependent kinases that influence gene expression, metabolism, and cytoskeletal dynamics (Bandyopadhyay et al., 2004, Maier and Bers, 2007). SOCE, initiated by ER Ca²⁺ store depletion, involves STIM1-Orai1 coupling to sustain Ca²⁺ influx at the PM, a mechanism vital for immune responses and prolonged signaling under stress (Hogan and Rao, 2015, Lee et al., 2020).

### Importance of PIP-Mediated Modulation of Ca²⁺ ATPase Activity in Health and Disease

Under conditions of intracellular Ca²⁺ overload, a pronounced interaction emerges between Ca²⁺ and PIPs, particularly PI(4,5)P_2_. Ca²⁺ binds to the anionic headgroups of PIP molecules, inducing their clustering into electrostatic complexes (Ellenbroek et al., 2011). Each PI(4,5)P_2_ molecule carries multiple negative charges (net charge of −3 or −4 at physiological pH), allowing Ca²⁺ to electrostatically bridge adjacent PIP headgroups, forming nanoscopic Ca²⁺-PI(4,5)P₂ clusters (Ellenbroek et al., 2011). This sequestration reduces the diffusibility of PI(4,5)P_2_ within the membrane, limiting its access to PIP-binding proteins (Simonsen et al., 2001).

Notably, beyond the general phenomenon of Ca²⁺-PI(4,5)P_2_ clustering, multiple research groups, including ours, have identified the specific molecular mechanisms underlying the direct interaction of Ca²⁺ with the phosphorylated headgroups of various PIPs, including PI(3,4)P_2_, PI(4,5)P_2_, and PI(3,4,5)P_3_. Through biochemical analysis, our group demonstrated that Ca²⁺ binds to 2 adjacent phosphate groups within PIPs, resulting in the formation of stable Ca²⁺-PIP complexes or clusters (Kang et al., 2017). This interaction effectively sequesters PIPs within membrane microdomains and inhibits their electrostatic association with the pleckstrin homology (PH) domains of key signaling proteins, including AKT, phospholipase C-delta, and insulin receptor substrate 1. Consequently, the Ca²⁺-PIP complex formation impairs the membrane localization of these PH domain-containing proteins, leading to the disruption of insulin signaling and positioning intracellular Ca²⁺ as a negative regulator of PIP-mediated signal transduction (Kang et al., 2017). This sequestration of PIPs by Ca²⁺ not only disrupts proximal signaling but also contributes to broader metabolic dysfunction. In hepatocytes, impaired membrane localization of AKT and insulin receptor substrate 1 attenuates PI3K-AKT signaling, leading to the dephosphorylation and nuclear accumulation of FOXO1 (Kang et al., 2017, Lee et al., 2023), which drives gluconeogenic gene expression (Taniguchi et al., 2006). Concurrently, diminished AKT activity fails to suppress SREBP1c, promoting lipogenesis and hepatic lipid accumulation. In adipocytes, AKT impairment reduces GLUT4 translocation to the PM, limiting glucose uptake and enhancing lipolysis (Taniguchi et al., 2006). These alterations result in systemic insulin resistance and metabolic inflexibility. Furthermore, AKT inactivation indirectly compromises SERCA function by preventing PLN phosphorylation (Catalucci et al., 2009), thereby perpetuating cytosolic Ca²⁺ overload. This establishes a deleterious feedback loop in which Ca²⁺-PIP complex formation reinforces Ca^2+^ dyshomeostasis and disrupts metabolic signaling. Complementary to our findings, Bilkova et al. (2017) employed molecular dynamics simulations to reveal that Ca²⁺ interacts directly with the phosphorylated headgroups of PI(4,5)P_2_, specifically restricting the membrane recruitment of phospholipase C-delta (Bilkova et al., 2017). Furthermore, elevated intracellular Ca²⁺ levels have been shown to prevent the membrane localization of PI3K-C2α, which contains a Ca²⁺-independent C2C domain, thereby further compromising PI3K signaling pathways (Chen et al., 2018). Collectively, these studies indicate that Ca²⁺-PIP complex formation universally disrupts the electrostatic interactions between PIPs and either the PH or C2 domains of signaling proteins via electrostatic repulsion. This interference hinders the proper membrane recruitment and function of these proteins, ultimately impairing insulin and PI3K signaling, and contributing to cellular dysfunction (Bilkova et al., 2017, Chen et al., 2018, Kang et al., 2017). Pharmacological interventions that restore intracellular Ca²⁺ homeostasis have demonstrated therapeutic potential. For example, the antihypertensive agent candesartan effectively reduces obesity-associated intracellular Ca²⁺ overload by upregulating the expression of Ca^2+^-ATPases, such as SERCA2 and PMCA2, in hepatocytes. Restoration of Ca^2+^ pump activity facilitates AKT membrane localization and enhances insulin sensitivity (Lee et al., 2023), underscoring the clinical relevance of targeting intracellular Ca²⁺ dynamics.

Ca^2+^-ATPases, specifically SERCA and PMCA, are indispensable in maintaining intracellular Ca^2+^ homeostasis (Fig. 4). Dysregulation of these pumps has been implicated in the pathophysiology of metabolic disorders, cardiovascular diseases, neurodegenerative diseases, and cancer (Oh, 2023, Tong et al., 2016). In metabolic tissues, SERCA dysfunction in pancreatic β-cells and hepatocytes impairs insulin signaling, lipid metabolism, and energy balance, thereby contributing to insulin resistance (Loncke et al., 2021, Missiroli et al., 2018). Moreover, defective ER-mitochondrial Ca^2+^ crosstalk in mitochondria-associated membranes exacerbates oxidative stress and lipotoxicity. In neurodegenerative diseases, such as Alzheimer’s and Parkinson’s disease, Ca^2+^-ATPase dysfunction results in ER stress (Le et al., 2025), mitochondrial Ca^2+^ overload, and neuronal death (Berrocal and Mata, 2023; Fujii et al., 2023; Hasan et al., 2024; He et al., 2025; Kim et al., 2022). Interestingly, submaximal inhibition or knockdown of SERCA extends lifespan in C. elegans (Romero-Sanz et al., 2023) via a mechanism independent of insulin or sirtuin pathways but dependent on functional mitochondria, AMPK activation, and mTOR inhibition (García-Casas et al., 2021). The effect likely involves reduced ER-mitochondrial Ca²⁺ transfer that triggers longevity-associated signaling. Cardiovascular diseases, including heart failure, are characterized by SERCA2a deficiency, which impairs cardiomyocyte contractility and contributes to vascular pathology (Frank et al., 2003; Giorgi et al., 2012; Kho, 2023; Loncke et al., 2021). Furthermore, in cancer, dysregulated Ca^2+^ signaling enhances mitochondrial bioenergetics and metabolic adaptability, supporting tumor progression (Finkel et al., 2015; Missiroli et al., 2018; Park et al., 2023).

Notably, PIPs regulate Ca^2+^-ATPase function in a context-dependent manner. Under physiological conditions, PI(4,5)P_2_ enhances PMCA activity by increasing its affinity for ATP- and Ca^2+^-bound calmodulin, thereby promoting Ca^2+^ extrusion (Moccia et al., 2023). However, during pathological Ca^2+^ overload, Ca²⁺-PI(4,5)P_2_ complexes may disrupt PMCA-PIP_2_ interactions (Oh, 2023), potentially impairing PMCA function and exacerbating cytosolic Ca^2+^ accumulation. Similarly, SERCA activity is modulated by AKT-mediated phosphorylation of phospholamban (PLN), which relieves its inhibitory effect and promotes Ca^2+^ reuptake in the ER (Catalucci et al., 2009, MacLennan and Kranias, 2003). However, increased intracellular Ca²⁺ levels hinder the membrane localization of AKT by promoting the formation of Ca²⁺-PIP complexes (Kang et al., 2017), potentially reducing PLN phosphorylation and impairing SERCA activity (Fig. 4). This creates a deleterious feedback loop, in which Ca^2+^ overload perpetuates SERCA and PMCA dysfunction, contributing to disease progression (Oh, 2023, Richardson et al., 2020). The chronic formation of Ca²⁺-PIP complexes may serve as a key pathological driver in metabolic diseases, cardiovascular conditions, and neurodegeneration. These findings highlight the dualistic role of PIPs, which function as facilitators of Ca^2+^ transport under physiological conditions but act as inhibitors under sustained Ca^2+^ stress. Therapeutic targeting of Ca²⁺-PIP interactions represents a promising strategy for restoring Ca^2+^ homeostasis and ameliorating disease-associated cellular dysfunction.

### Therapeutic Potential of Targeting Ca²⁺-ATPases: SERCA and PMCA as Key Modulators

PMCAs and SERCAs are fundamental components in maintaining intracellular Ca^2+^ homeostasis. Accumulating evidence supports their involvement in various pathologies. Targeting these pumps using selective inhibitors or activators has expanded our understanding of Ca^2+^ signaling mechanisms and has highlighted their potential for clinical applications (Alexander et al., 2015; Chaudhary et al., 2001; Doan et al., 2015; Lee et al., 2023; Pande et al., 2006; Shah et al., 2005; Souto-Guevara et al., 2024; Szewczyk et al., 2010) (Table 1).

**Table 1:** Pharmacological modulators of Ca^2+^-ATPases

Target	Inhibitors	Activators	Associated diseases	References
PMCAs	Caloxin 2A1, PMCA1 selective	-	Neurological disorders, cancer	(Chaudhary et al., 2001)
	Caloxin 1b3, PMCA1 selective	-	Hypertension, neurodegeneration	(Szewczyk et al., 2010)
	Caloxin 1b1, PMCA4 selective	-	Cardiovascular disease	(Pande et al., 2006)
	Aurintricarboxylic acid	-	Cancer, neuroinflammation	(Souto-Guevara et al., 2024)
SERCAs	Thapsigargin	Ochratoxin A (nephrotoxicity)	Prostate cancer (via mipsagargin)	(Alexander et al., 2015)
	Cyclopiazonic acid		Research reagents (nontherapeutic)	(Alexander et al., 2015)
	Butylhydroquinone		ER stress modeling, cancer	(Alexander et al., 2015)
	Mipsagargin		Hepatocellular carcinoma	(Doan et al., 2015)
		Rosiglitazone	Type 2 diabetes, insulin resistance	(Shah et al., 2005)
		Candesartan	Type 2 diabetes, insulin resistance	(Lee et al., 2023)

PMCAs, plasma membrane Ca^2+^-transporting ATPases; SERCAs, sarcoplasmic/endoplasmic reticulum Ca^2+^-ATPases.

SERCA plays an essential role in transporting cytosolic Ca²⁺ into the SR and ER, thereby regulating processes, including muscle contraction, relaxation, and metabolic functions (Tadini-Buoninsegni et al., 2018, Vangheluwe et al., 2005). Aberrant SERCA function has been implicated in various pathologies, including heart failure, diabetes, and muscular disorders. To restore proper Ca^2+^ dynamics, therapeutic strategies have focused on SERCA activators that enhance pump activity and improve Ca^2+^ sequestration. Notably, small molecules, such as CDN1163, have demonstrated efficacy in stabilizing SERCA conformation and increasing ATPase activity, resulting in improved cardiac function and Ca^2+^ handling (Xu and Van Remmen, 2021). Beyond their cardiovascular benefits, SERCA activators have shown promise in ameliorating skeletal muscle dysfunction, reducing Ca^2+^-induced cytotoxicity in muscular dystrophies, and modulating energy metabolism in metabolic diseases (Aureliano et al., 2022). CDN1163 serves as a pharmacological proof-of-concept for SERCA2a activation in heart failure, diabetes, and MASH (Kho et al., 2015). However, its poor physicochemical properties, lack of structure-guided SAR optimization, and absence of clinical progression limit its translational potential. Future strategies should focus on next-generation SERCA2a modulators with improved specificity, such as DWORF-based peptides or nanoparticle-mediated delivery. Chemoproteomic profiling of CDN1163 derivatives may aid in identifying off-target effects and inform the development of safer, more effective analogs.

Conversely, PMCA is responsible for the extrusion of Ca^2+^ from the cytoplasm to the extracellular space, ensuring low intracellular Ca^2+^ concentrations. The dysregulation of PMCA has been linked to cancer progression, implicating PMCA a novel target for anticancer therapies. PMCA inhibition disrupts Ca^2+^ homeostasis in cancer cells, leading to increased intracellular Ca^2+^ levels and apoptosis (Fujii et al., 2023; Richardson et al., 2020). Polyoxotungstates, including the Preyssler-type polyoxotungstate (P₅W₃₀), have been identified as potent and selective PMCA inhibitors with demonstrated efficacy against various cancers, including breast, prostate, and colon cancer (Aureliano et al., 2022, Peters et al., 2016). Furthermore, these inhibitors can potentiate existing cancer therapies and combat drug resistance (Curry et al., 2016, Park et al., 2023, Richardson et al., 2020). However, the context-dependent role of PMCA as both a cell survival mediator and trigger for cell death necessitates a nuanced understanding of its regulation by cellular energy status and cofactors (Peters et al., 2016).

A crucial yet often underappreciated regulatory mechanism of Ca²⁺-ATPase function involves PIP interactions. Specifically, PI(4,5)P_2_ acts as an essential cofactor for PMCA, enhancing pump activity and the depletion-inhibiting function. Additionally, PI(3,4,5)P_3_-mediated kinase signaling pathways upregulate SERCA expression and activity. These lipid-mediated regulatory mechanisms ensure the dynamic responsiveness of Ca^2+^ pumps to cellular stimuli and stress. Importantly, perturbation of PIP-pump interactions, particularly under conditions of intracellular Ca²⁺ overload, can compromise pump functionality and contribute to cellular injury and disease progression. Therapeutic strategies aimed at stabilizing favorable PIP-pump interactions or pharmacologically mimicking their effects hold considerable promise for correcting Ca^2+^ dysregulation in various diseases. Continued research into the molecular intricacies of lipid-ion pump interplay is essential for translating these findings into clinically viable interventions. The conceptual framework of “lipid regulation of ion pumps” represents a fertile ground for developing targeted therapies to restore Ca^2+^ homeostasis and mitigate disease pathology.

### Technological Advances in Ca^2+^-ATPase Research: Structure, Screening, and Imaging

Recent technological advancements have greatly expanded our understanding of Ca^2+^-ATPases, encompassing their structural dynamics, functional mechanisms, and regulatory roles. This review highlights 3 major areas of progress: structural analysis using cryo-EM and X-ray crystallography, high-throughput screening (HTS) for identifying Ca^2+^-ATPase modulators, and advanced imaging techniques for studying intracellular Ca^2+^ dynamics. These advancements are reshaping studies on Ca^2+^-ATPases and their critical functions in cellular physiology and pathology.

The advent of high-resolution cryo-EM has transformed our ability to study the structural dynamics of Ca^2+^-ATPases. One landmark discovery is the 3.3-Å resolution cryo-EM structure of human SERCA2b in the E1·2Ca²⁺ state (Courbon and Rubinstein, 2022). This analysis revealed a novel conformation characterized by a closer arrangement of the cytosolic domains than previously resolved crystal structures (Zhang et al., 2021), underscoring the dynamic nature of SERCA2b domains and their role in ATP binding and Ca²⁺ transport. These structural insights are pivotal for understanding intracellular Ca^2+^ regulation and its implications in cellular processes. For example, the conformational state of SERCA2b during ATP binding and Ca²⁺ transport provides valuable information on how Ca^2+^ overload disrupts the localization and function of PIP-binding proteins. By modulating SERCA2b conformational transitions, researchers may identify strategies to mitigate the adverse effects of Ca^2+^ dysregulation, such as those linked to PIP signaling imbalances. The integration of cryo-EM with other structural techniques, such as X-ray crystallography, has further enriched our knowledge of the molecular mechanisms of Ca^2+^-ATPases. Collectively, these techniques reveal the intricate conformational changes that occur during Ca^2+^ transport, and offer novel insights into the design of targeted therapeutics for Ca²⁺-related disorders.

HTS technologies have significantly accelerated the discovery of small-molecule modulators of Ca^2+^-ATPases, particularly those with therapeutic potential. HTS platforms, including fluorescence resonance energy transfer-based biosensors and NADH-coupled ATPase assays, are instrumental in identifying modulators of enzymes, such as SERCA2a (Bidwell et al., 2022, Roopnarine et al., 2023, Schaaf et al., 2020). These approaches assess Ca^2+^-ATPase activity, Ca^2+^ transport, and protein-protein interactions, such as those between SERCA2a and PLN, to screen for activators or inhibitors. Secondary assays have further validated HTS hits by evaluating their effects on Ca^2+^ uptake, isoform specificity, and functional outcomes in tissues, such as cardiac and skeletal muscles. In addition, fluorescent biosensors, which improve precision by minimizing false positives, have proven invaluable for characterizing the pharmacological properties of candidate compounds. HTS technologies are paving the way for drug discovery efforts targeting diseases linked to Ca^2+^ dysregulation, including heart failure and muscle dysfunction. As our understanding of Ca^2+^-ATPase modulators evolves, future research will likely focus on optimizing the selectivity, efficacy, and safety of these agents, as well as exploring combination therapies that target multiple Ca^2+^-regulating pathways.

Recent advancements in Ca^2+^ imaging techniques have revolutionized the study of intracellular Ca^2+^ dynamics and the role of Ca^2+^-ATPases in cellular signaling. Additionally, the recent development of a novel genetically encoded ratiometric ER Ca^2+^ indicator designated GFP-RCEPIA1er further enhances our ability to rapidly quantify ER Ca²⁺ concentrations and directly assess SERCA activity in live cells. This approach facilitates the precise characterization of regulatory micropeptides, such as PLN, and enables HTS of therapeutic molecules targeting SERCA (Cunningham et al., 2025). This powerful platform can address Ca^2+^ dysregulation in cardiovascular and neurodegenerative diseases. Genetically encoded Ca^2+^ indicators, such as GCaMP6, are indispensable tools for tracking Ca^2+^ activity in intact tissues and subcellular compartments (Tian et al., 2009). These indicators are optimized for in vivo imaging, enabling real-time monitoring of neuronal activity in large cell populations and a finer resolution of subcellular processes. High-throughput fluorescence imaging techniques, such as those using Cal-520 dye, have further enhanced the sensitivity and brightness of Ca^2+^ measurements in primary neuronal cultures (Sasaki et al., 2024). These methods facilitate the assessment of Ca^2+^ transients, enabling the evaluation of the seizure risk of compounds during early drug discovery and identification of neuroprotective agents for conditions such as stroke. Ex vivo imaging in model organisms, such as Drosophila, has also provided new opportunities to study Ca^2+^ signaling dynamics in live tissues (Dana et al., 2019). This approach revealed the critical roles of Ca^2+^ signaling in processes that include cell differentiation, immune responses, and wound healing. Together, these imaging advances provide unprecedented insights into the spatial and temporal dynamics of Ca^2+^ signaling in health and disease.

Technological advancements in structural analysis, HTS, and Ca^2+^ imaging have profoundly enhanced our understanding of Ca^2+^-ATPases. These tools reveal the molecular mechanisms underlying Ca^2+^ transport and enable the development of novel therapeutic strategies. Cryo-EM and X-ray crystallography provide structural blueprints for understanding the effects of Ca^2+^ dysregulation on cellular processes, and offer potential intervention points. HTS platforms are driving the discovery of small-molecule modulators that target Ca^2+^-ATPases with high precision, paving the way for the effective treatment of Ca^2+^-related diseases. Advances in Ca^2+^-imaging techniques have bridged the gap between molecular mechanisms and physiological outcomes, allowing researchers to connect intracellular Ca^2+^ dynamics to broader cellular functions. As these technologies continue to evolve, they hold immense promise for uncovering new therapeutic opportunities and enhancing our understanding of Ca^2+^ signaling in health and disease. Future research should prioritize integrating these methodologies to explore the interplay between the structural dynamics, regulatory mechanisms, and physiological functions of Ca²⁺-ATPases. Additionally, the optimization of drug discovery pipelines through advanced imaging and HTS methods will be crucial for translating these findings into clinical applications. Collectively, these advancements will drive continued progress in understanding and targeting Ca^2+^-ATPases for therapeutic benefits.

## CONCLUSIONS AND PERSPECTIVES

This review explores the intricate dynamics of Ca²⁺ homeostasis and the pivotal role of PIPs in regulating cellular signaling across a multitude of pathways. PIPs are a family of 7 interconvertible phospholipids found in mammalian cells. These phospholipids are critical for the recruitment of proteins harboring PH domains to the PM. This action is crucial for regulating various cellular functions, including cell growth, survival, vesicular trafficking, and cytoskeletal reorganization. The PI3K/AKT signaling pathway is a central route activated by stimuli such as growth factors, cytokines, and cellular stress. This pathway is involved in the phosphorylation of PI(4,5)P_2_ to PI(3,4,5)P_3_ by PI3K. This phosphorylation event facilitates the recruitment and activation of PH domain-containing proteins, such as PDK, AKT, and GTPases, which are essential for processes, including cell survival, proliferation, and metabolism.

The maintenance of Ca²⁺ homeostasis is equally critical, mediated by transporters and pumps such as PMCAs, SERCAs, and NCLX. These proteins are integral to sustaining appropriate Ca²⁺ levels within the ER and mitochondria, which are vital for cellular metabolism and signaling. SERCAs and PMCAs play pivotal roles in pumping Ca²⁺ into the ER and out of the cell, respectively. Dysregulation of these Ca²⁺ transport mechanisms can lead to increased intracellular Ca²⁺ levels and the disruption of Ca²⁺ homeostasis, contributing to the pathology of various metabolic diseases, including diabetes, cardiovascular diseases, and obesity.

The coupling of PIPs with intracellular Ca²⁺ signaling is of particular importance due to the significant impact of intracellular Ca²⁺ dysregulation in numerous pathological conditions. A novel aspect of this coupling is the Ca²⁺-mediated inhibition of membrane localization of PIP-binding proteins, suggesting a regulatory mechanism that is sensitive to changes in intracellular Ca²⁺ levels. This mechanism influences diverse cellular functions, such as endocytosis, intracellular trafficking, and gene expression, offering new insights into the complex interplay between PIPs and intracellular Ca²⁺ signaling.

Understanding these intricate relationships is crucial for developing therapeutic strategies to treat metabolic diseases. However, given the complexity and dynamism of intracellular Ca²⁺ and PIP signaling, any therapeutic interventions targeting these pathways should be approached with caution. The potential risks and benefits must be carefully evaluated to prevent unintended consequences, particularly in patients with obesity, diabetes, or other chronic metabolic disorders. Although the prospects for targeting these pathways are promising, they underscore the need for meticulous research and clinical trials to ascertain their safe and effective therapeutic applications.

## ETHICS APPROVAL AND CONSENT TO PARTICIPATE

Not applicable.

## FUNDING AND SUPPORT

This work was supported by grants from the National Research Foundation of Korea funded by the Korean government (MSIT) (NRF-2021R1A5A2030333 and RS-2024-0035412012982076870101) and the Gachon University Research Fund (GCU-202008430007).

## CRediT Authorship Contribution Statement

**Seung Wan Noh:** Writing – Review and editing, Writing – Original draft, Investigation. **Ok-Hee Kim:** Writing – Review and editing, Writing – Original draft, Funding acquisition, Formal analysis, Conceptualization. **Byung-Chul Oh:** Writing – Review and editing, Writing – Original draft, Visualization, Project administration, Funding acquisition, Formal analysis, Conceptualization. **Hyun-Oh Gu:** Writing – Review and editing, Writing – Original draft, Investigation.

## DECLARATION OF COMPETING INTERESTS

The authors declare that they have no competing interests.
